# Benthic studies adjacent to Sakhalin Island, Russia, 2015 I: benthic biomass and community structure in the nearshore gray whale feeding area

**DOI:** 10.1007/s10661-022-10017-8

**Published:** 2022-10-18

**Authors:** Arny L. Blanchard, Natalia L. Demchenko, Lise A. M. Aerts, Sergei B. Yazvenko, Victor V. Ivin, Ilya A. Shcherbakov

**Affiliations:** 1 Blanchard, Ecological, North Pole, AK 99705 USA; 2 A.V. Zhirmunsky National Science Center of Marine Biology, Far East Branch of Russian Academy of Sciences, Vladivostok, Russia; 3 LAMA Ecological, Anchorage, AK 99502 USA; 4 LGL Limited, Sidney, BC V8L 3Y8 Canada; 5 L.S. Berg State Research Institute On Lake and River Fisheries, Saint Petersburg, Russia

**Keywords:** Benthic ecology, Marine ecology, Ecosystem variability, Macrobenthos, Sea of Okhotsk

## Abstract

Okhotsk or western gray whales feed in summer along the northeastern coast of Sakhalin Island, Russia, a region with oil and gas extraction facilities. Seismic surveys increased sound levels in the nearshore feeding area in 2015 for part of the summer, potentially displacing whales from preferred foraging habitat or reducing foraging efficiency. Since lost foraging opportunities might lead to vital rate effects on this endangered species, detailed benthic surveys were conducted to characterize benthic community biomass patterns and spatial and temporal differences. Benthic biomass demonstrated strong spatial–temporal interactions indicating that prey biomass differences among locations were dependent on sampling period. Of greatest interest, Amphipoda biomass declined from June to October in the northern and southern portions of the nearshore study area but increased in the middle and Actinopterygii biomass increased in the northern area in mid-summer. Water depth and sediment type were significant covariates with community structure, and water depth strongly covaried with bivalve biomass. Total average prey biomass was ~ 100 g/m^2^ within the nearshore feeding area with no evidence of reduced biomass among sampling periods or locations, although there were fewer amphipods in the south. Multi-prey investigations provide a stronger basis for inferences than single-prey studies of amphipods when gray whales feed on diverse prey. Benthic community-level variability was moderate to high as would be expected for a shallow-water nearshore area. Overall, spatial and temporal changes in dominant macrofauna biomass reflected small to medium-sized effects that were well within the natural boundaries expected for benthic communities.

**Supplementary Information:**

The online version contains supplementary material available at 10.1007/s10661-022-10017-8.

## Introduction

Okhotsk or western gray whales (*Eschrichtius robustus*), a small population consisting of less than 200 individuals (Cooke et al., 2017), rely largely on biomass-rich benthic prey in and near the offshore oil and gas fields adjacent to northeastern Sakhalin Island, Russia (Demchenko, 2010; Demchenko & Fadeev, 2011; Demchenko et al., 2016; Sobolevskii et al., 2000; Weller et al., 1999, 2002). Underwater sounds associated with industry activities (including sounds from vessel traffic, seismic surveys, and drilling) may alter whale behaviors, possibly leading to cessation of feeding and movement away from prime feeding habitats (Bröker et al., 2015; Gailey et al., 2007, 2016; Muir et al., 2015; Villegas-Amtmann et al., 2017; Yazvenko et al., 2007). This might, in turn, lead to changes in whale energetic balances if behavioral changes are severe or persistent (Villegas-Amtmann et al., 2015, 2017) and prey resources are distributed unequally across the marine landscape. Increased sound levels in the marine environment during seismic surveys (ensonification) is a short-term stressor but effects may persist in the presence of overlapping and synergistic changes in other ecosystem elements, such as prey distributions (Blanchard et al., 2017; Crain et al., 2008; Harwell et al., 2010; Peterson, 2001).

Declining biomass in the Sakhalin Island feeding areas reflects broader ecological and climatic changes in the Sea of Okhotsk and is of concern for western gray whale population success (Blanchard et al., 2019; IUCN, 2019). Benthic communities in the feeding areas are not expected to experience measurable impacts from seismic surveys, but the communities are changing due to other factors, as indicated by correlations of macrobenthic biomass with climate variables and as noted for other coastal systems (Blanchard, 2015; Blanchard et al., 2010; Cloern et al., 2010). Prey resource limitations are a challenge for gray whales (Coyle et al., 2007; IUCN, 2019; Moore, 2008), potentially amplifying the importance of behavioral responses to anthropogenic disturbance. Thus, mitigation of effects from seismic surveys on western gray whales requires, in part, an understanding of the dynamics of prey resources.

Here, we test the hypotheses that benthic biomass and community characteristics varied temporally across three summer and fall sampling periods and spatially within the gray whale nearshore feeding area. Univariate statistical hypotheses were evaluated with ANCOVA to understand the spatial and temporal dynamics of biomass for dominant macrobenthic fauna. Multivariate analyses were also conducted to characterize community biomass patterns. The statistical analyses provide insights into the sources of variability driving benthic biomass and community structure in the nearshore feeding area. This work contributes to a larger effort aimed at understanding impacts on western gray whales associated with seismic surveys during the 2015 summer and fall seasons (Aerts et al. 2022). The benthic component includes three additional papers discussing long-term spatial–temporal trends in the benthic community (Blanchard et al., 2019), energy density of the benthic prey (Maresh et al. 2022), and spatial regression modeling of dominant macrobenthos (Blanchard et al. 2022).

## Materials and methods

### Study area

**Fig. 1:**
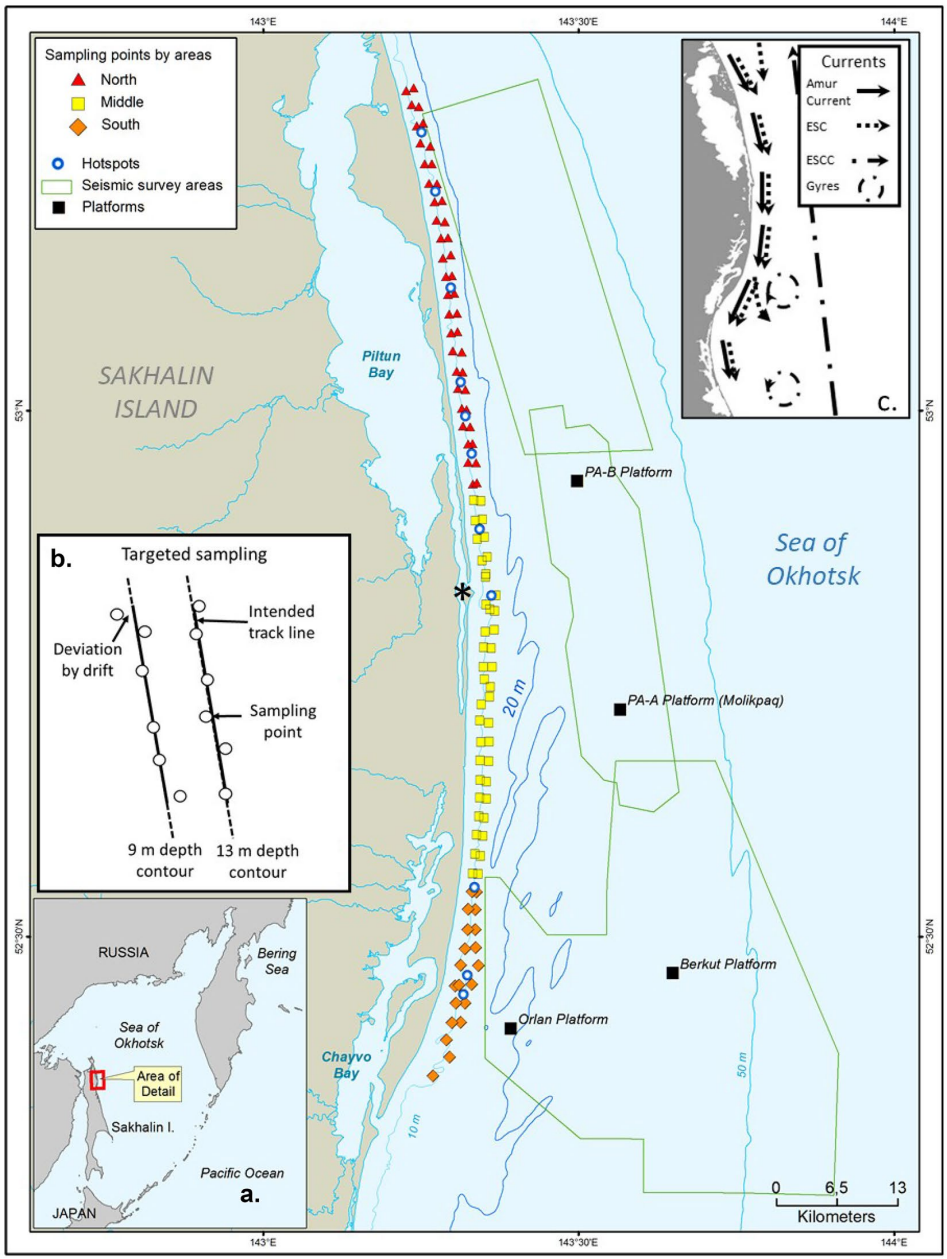
The 2015 benthic sampling locations within the Sakhalin Island gray whale nearshore feeding area, Russia. Inset a gives the geographic reference of the gray whale feeding area, inset b the design for targeted sampling, and inset c the major circulation patterns. The * marks the mouth of Piltun Bay. In inset c, Amur current, waters from the Amur River; ESC, the East Sakhalin Current; and ESCC, the East Sakhalin counter-current

The western gray whale feeding area is located off the northeastern coast of Sakhalin Island, Russia (Fig. 1). An area of 200 km^2^ (100 km long by 2 km wide) within the nearshore feeding area was sampled for macrobenthos. The study area’s hydrography is influenced by brackish water from the Amur River via circulation around the northern tip of Sakhalin Island, outflows from Piltun Bay along the eastern shoreline, and winter ice cover (Rutenko & Sosnin, 2014; SEIC, 2003; Shevchenko & Chastikov, 2008). Wind-driven upwelling of nutrient-enriched water from the Sea of Okhotsk contributes to biological productivity in summer, but wind-driven turbulence and strong southerly currents maintain a mosaic of mobile substrates (Rutenko et al., 2009; SEIC, 2003). Winter oceanographic characteristics are controlled by the Eastern Sakhalin Current that flows southward through the nearshore region of Sakhalin Island and the northeastern Sakhalin polynya (Ebuchi, 2006; Nihashi et al., 2011; Shevchenko & Chastikov, 2008). Sediment metals and hydrocarbons are reported to be at background levels, although some seawater contamination may be present with the Amur River a possible contributing source (Jen, 2003; Leonov et al., 2010; Levshina et al., 2009; Lukyanova et al., 2014; SEIC, 2003). Western gray whales in the Sakhalin Island nearshore feeding area primarily prey on amphipods (mostly *Monoporeia affinis* and *Eogammarus schmidti*) but also feed on the isopods *Saduria entomon* and *Synidotea cinerea* and the sand lance *Ammodytes hexapterus* (Blanchard et al., 2019; Budnikova & Blohkin, 2012; Demchenko, 2010; Fadeev, 2011; Sobolevskii et al., 2000; Zimushko & Lenskaya, 1970).

### Sampling design

Macrofauna and sediment samples were collected from the R/V *Igor Maksimov* with a 0.2-m^2^ van Veen grab. The draft of the research vessel limited safe operations to water deeper than 9 m although some depths of 7 m were sampled. Onboard, 3 replicate grab samples were collected per station and rinsed over a series of nested sieves with 5.0, 1.0, and 0.5-mm mesh screens to capture macrofaunal organisms. Residues were preserved using a 4% formalin solution. In the laboratory, animals were sorted from the sediment residues, identified to appropriate class of higher taxonomic categories, counted, and weighed. Sediment characteristics were visually observed and qualitatively estimated for one sample collected at each station during sampling. Sediments were categorized by visually dominant sediment characteristics as medium or fine sand. Where stations overlapped with historical survey sampling (Blanchard et al., 2019), visual sediment classifications were linked to granulometric data with medium sand reflecting grain-sizes > 0.25 mm and fine sands < 0.25 mm.

Sampling in the nearshore gray whale feeding area was designed to characterize spatial and temporal (within summer to fall) variations of benthic biomass. The detailed grid sampling design consisted of two lines parallel to the coast following the 9 m and 13 m isobaths and spaced ~ 2 km apart in an east–west direction (Fig. 1). Planned sampling locations were positioned at ~ 2 km intervals along each of these two lines and consisted of 52 sampling stations at both the 9 m and 13 m isobaths. The grid was divided into North, Middle, and South Zones with the extent of each zone roughly matching seismic survey project boundaries and associated areas of ensonification that might influence gray whale feeding (Fig. 1; Aerts et al. 2022). The three 2015 benthic sampling periods were early season (Period 1; 19 June to 7 July) with 68 stations sampled; middle season (Period 2; 24 July to 19 August) with 101 stations sampled; and late season (Period 3; 14 September to 24 October) with 54 stations sampled. The sampling periods roughly align with whale presence: few gray whales are observed early in the summer prior to July; whales are most numerous in mid-summer; and whale numbers decline during late summer and fall. The entire detailed grid could not be completed during Periods 1 and 3, so stations along the 9 m and 13 m lines were sampled in a staggered pattern to allow for even coverage across the entire sampling grid. The number of stations sampled in each zone and period were Period 1, North Zone = 22, Middle Zone = 32, and South Zone = 14 stations; Period 2, North Zone = 42, Middle Zone = 41, and Zone S = 18 stations; and Period 3, North Zone = 21, Middle Zone = 21, and South Zone = 12 stations.

Benthic communities were further characterized through targeted sampling conducted in areas where whales were observed to intensively and/or persistently feed (as determined by shore-based biologists) and are called feeding hotspots (Fig. 1, inset b). The vessel traveled along 2 transects that were 2 km long at the 9 m and 13 m isobaths (the two north–south lines in Fig. 1, inset b) and collected 12 individual grabs distributed along the vessel’s course. The spatial scale of sampling within each area was small enough to consider each grab to be a replicate for the analyses. Feeding hotspots were sampled in between Periods 1 and 2. The number of feeding hotspots sampled in each zone was North Zone = 6, Middle Zone = 2, and South Zone = 3. Where whale feeding activities were in water shallower than the safety limits of the research vessel, the sampling area was located as close as possible to the area of interest.

### Statistical methods

Analyses relied on descriptive, univariate, and multivariate statistical methods for community analysis. Wet tissue biomass (g/m^2^) was determined for macrofaunal groups with a focus on characteristic animals including Actinopterygii (primarily the sand lance *Ammodytes hexapterus*), Amphipoda, Bivalvia, Cumacea, Echinoidea (primarily the sand dollar *Echinarachnius parma*), Isopoda, and Polychaeta. Biomass of characteristic animals (prey biomass) was calculated as the sum of the animals known to occur in gray whale diets and potential prey: Actinopterygii, Amphipoda, Bivalvia, Cumacea, Isopoda, and Polychaeta. Detailed summaries are presented in Supplementary Material Appendix A.

The percentage of amphipod biomass concentrations less than 60, 100, and 200 g/m^2^ wet weight biomass was calculated by period and zone to investigate seasonal differences in biomass. A total of 60 g/m^2^ wet weight was used as the lower cutoff for Amphipoda biomass to represent a rough minimum biomass for gray whale feeding based on prior literature (Coyle et al., 2007; Feder et al., 1994; Highsmith & Coyle, 1990, 1992; data transformed from dry to wet weight, as appropriate) and available data (Blanchard & Feder, 2014; Blanchard et al., 2013). Two additional cutoff values of 100 and 200 g/m^2^ were chosen with the cutoff value of 200 g/m^2^ selected to capture extreme high biomass values.

Analysis of covariance (ANCOVA) was conducted with mixed models to test for differences among sampling periods, sampling zones, and water depth for the detailed sampling grid. Mixed models included the random effect of station for each of the characteristic fauna and total biomass. Replicates were nested within the station effect to account for within-station error. Factors for analysis were zone and sampling period. Depth was included as a continuous predictor for ANCOVA, standardized to prevent collinearity, and included as a squared term to test for nonlinear responses. Biomass concentrations were *ln*(X + 1)-transformed to better meet ANOVA assumptions, and particularly that of equal variance for positive biomass values (biomass > 0). Residual plots were used to evaluate ANOVA assumptions and determine best corrections for assumption violations. Actinopterygii had many zero biomass values, but the sample size was large enough for an F-test and positive biomass values did not violate assumptions with transformation. Denominator degrees of freedom of mixed models were determined using the Satterthwaite approximation.

Minimum-effects (ME) hypotheses were applied to ANCOVA F-statistics as extensions of power analysis based on noncentral F-distributions (Cohen, 1988; Murphy et al., 2014). Effect sizes (ES) are a function of the noncentrality parameter $\lambda$ and are chosen to reflect meaningful levels of change (measured by the effect size *f* and associated percent variance (PV)). The ME approach evaluates the ME hypotheses that an observed effect is less than a chosen effect-size *f* (H_ME_: *f*_Obs_ < *f*_ME_ _ES_) and the alternative that the effect is greater than *f* (Ha: *f*_Obs_ ≥ *f*_ME_ _ES_). Statistical tests are conducted by comparing observed F-values (F_obs_) against noncentral F-distributions based on the selected effect-sizes, as opposed to comparison against a F-statistic under the null hypothesis with $\lambda$ = 0 (the hypothesis of no difference). Thus, a ME hypothesis is structured as an interval hypothesis where an interval with limits determined by the ES in a noncentral F-statistic defines the test hypotheses: H_ME_: F_obs_ ≤ F_Crit_ _ME_ _ES_ and the alternative Ha: F_obs_ > F_Crit_ _ME_ _ES_ where Crit ME ES = the ME hypothesis ES test critical value. λ can be approximated for a test using the equation $\lambda$=DF_Err_*PV/(1/PV) where *DF*_*er*r_ is the error (denominator) degrees of freedom from the ANCOVA for a specific effect (Murphy et al., 2014). The approximation is appropriate with a large sample size, as in the present study. For marine benthic studies, effects sizes of small (*f*_Small_ = 0.2, PV ≈ 4%), medium (*f*_Medium_ = 0.5, PV ≈ 23%), and large (*f*_Large_ = 0.8, PV ≈ 40%) have been proposed for chemical and physical disturbance to benthic communities and are used here (Blanchard et al., 2002). Post hoc power analysis indicated low power for the design with an F-statistic of 2 (~ median F-statistic for the observed depth^2^ effect), moderate power for F-values of 8 (~ median F for period and zone), and higher power for F = 16 (~ median F for depth). A power analysis and the complete set of ME hypothesis tests are presented in the Supplementary Material Appendix A.

Nonmetric multidimensional scaling (MDS) was applied to determine community trends. Biomass concentrations were *ln*(X + 1)-transformed prior to calculation of Bray–Curtis similarity coefficients using the community biomass matrix with rare groups excluded (Bray & Curtis, 1957). MDS was then conducted on the Bray–Curtis similarity matrix. Biomass values were averaged by station for multivariate analysis. Nonparametric, permutational multivariate analysis of variance (NPMANOVA) was conducted with period, zone, sediment type (fine and medium), and water depth (9 m and 13 m) as factors. Multiple comparisons following the NPMANOVA were performed with the Holm adjustment to *p*-values (α = 0.05).

All statistical analyses were conducted using R statistical software (ver 3.4.1; R Core Team, 2019). Mixed modeling was conducted using the *lme* function in the nlme library package (Pinheiro et al., 2020). MDS was conducted using the *metaMDS* function and NPMANOVA using the *adonis* function of the vegan library in R (Oksanen et al., 2017). Holm-adjusted multiple comparisons following the NPMANOVA were performed using the *pairwise.perm.t.test* function in the RVAideMemoire library (Hervé, 2017). Noncentral F-statistic critical values and ME hypothesis *p*-values were determined in R.

## Results

**Fig. 2:**
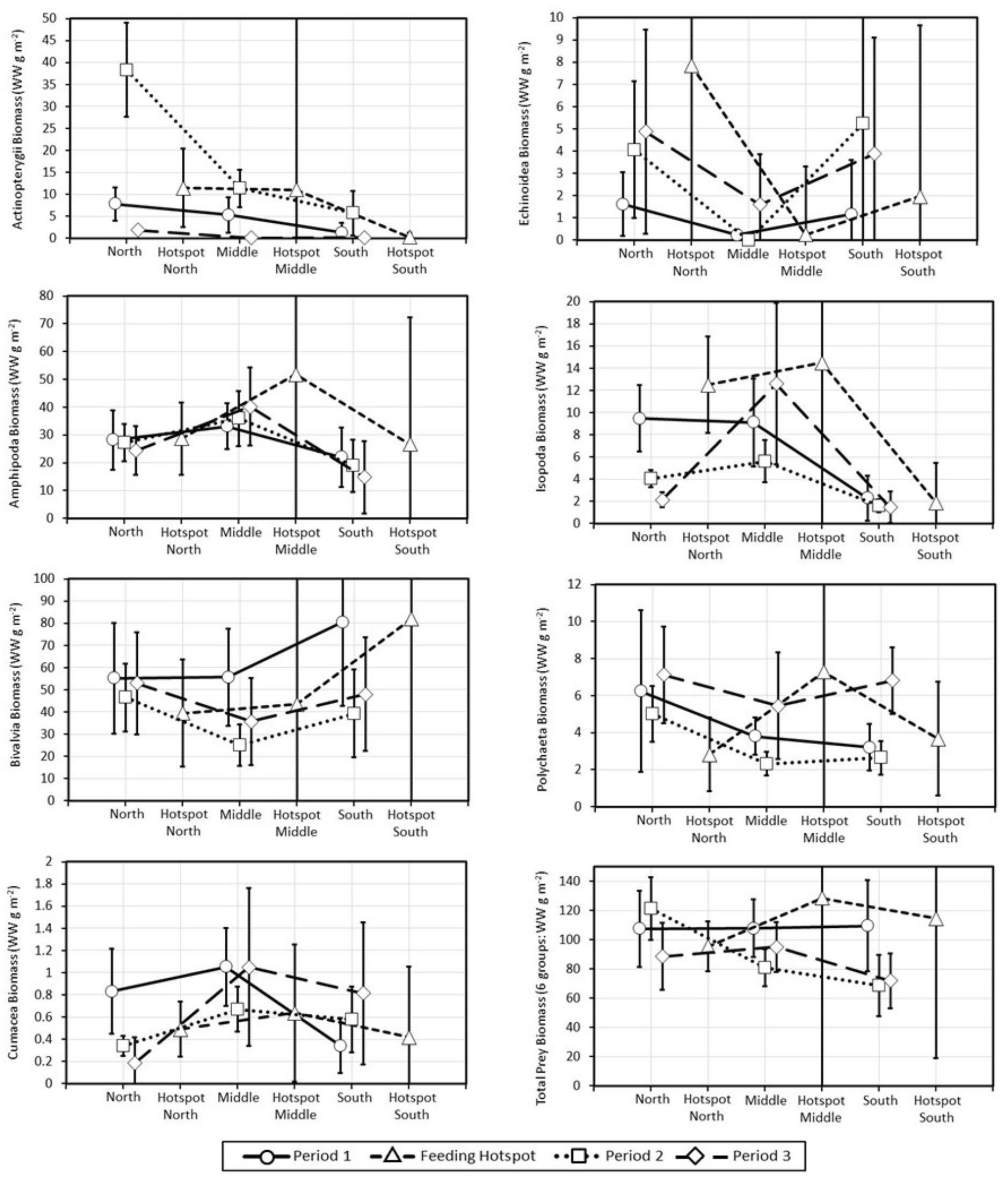
Average wet tissue biomass (g/m^2^) and 95% confidence intervals of Actinopterygii, Amphipoda, Bivalvia, Cumacea, Echinoidea, Isopoda, Polychaeta, and total prey biomass from the 2015 detailed grid and feeding hotspots. North, Middle, and South are the zones in the detailed grid. Feeding hotspots were sampled between Period 1 and 2. The very large confidence interval for Hotspot Middle is due to the small number of sites sampled in that zone (2). Data points for different periods within each zone are staggered for clarity

Biomass demonstrated significant spatial and/or temporal variability for each group (Table 1). The transformed biomass of every group differed by period or the zone*period interaction with depth or depth^2^ a significant predictor for all groups except Actinopterygii and Isopoda. There were two biomass patterns that stood out: (i) Amphipoda biomass increased over time in the Middle Zone but declined over time in the North and South Zones (reflected in the significant zone*period interaction), and (ii) Actinopterygii biomass concentrations demonstrated a very high peak in the North Zone during Period 2 (Fig. 2). Total biomass demonstrated some differences of interest in that it was constant across zones in Period 1, declined from the North to South Zone in Period 2, and declined slightly in Period 3 in the South Zone, although many confidence intervals overlapped. Average biomass values for feeding hotspots generally overlapped with the confidence intervals for the detailed grid, and feeding hotspot biomass values followed trends in surrounding areas. Despite the lower values in the Middle and South Zones in Periods 2 and 3, average total biomass was substantially higher in feeding hotspots in the Middle and South Zones, indicating the presence of high biomass patches. Most ANCOVA model effects were negligible to small-sized with F-statistics for 10 comparisons being larger than the ME hypothesis critical values for a small effect and only one, depth for Bivalvia, large enough to reject the ME hypothesis for a medium-sized effect.

**Table 1:** ANCOVA results for seven faunal categories and total biomass for the 2015 detailed grid. P-values in bold are significant at w = 0.05 (*p* ≤ 0.05)

Group	Zone	Period	Zone:period	Depth	Depth^2^
Actinopterygii	< 0.0001*	< 0.0001*	** < 0.0001**	0.3239	0.1497
Amphipoda	< 0.0001*	**0.0116**	**0.0079**	< 0.0001*	** < 0.0001**
Bivalvia	**0.0353**	**0.0000**	**0.0036**	< 0.0001**	0.7282
Cumacea	< 0.0001*	0.5969	< 0.0001*	0.1354	** < 0.0001**
Echinoidea	**0.0027**	**0.0148**	0.1835	< 0.0001*	0.0063
Isopoda	< 0.0001*	**0.0003**	< 0.0001*	0.4182	0.1748
Polychaeta	**0.0171**	** < 0.0001**	0.0502	< 0.0001*	0.3767
Total Prey	**0.0266**	**0.0010**	** < 0.0001**	** < 0.0001**	0.8119

An asterisk indicates a small effect (*f* = 0.2 representing natural variability) for a minimum-effects (ME) hypothesis and a double asterisk denotes a medium-sized effect (*f* = 0.5 representing transition across a boundary)

**Fig. 3:**
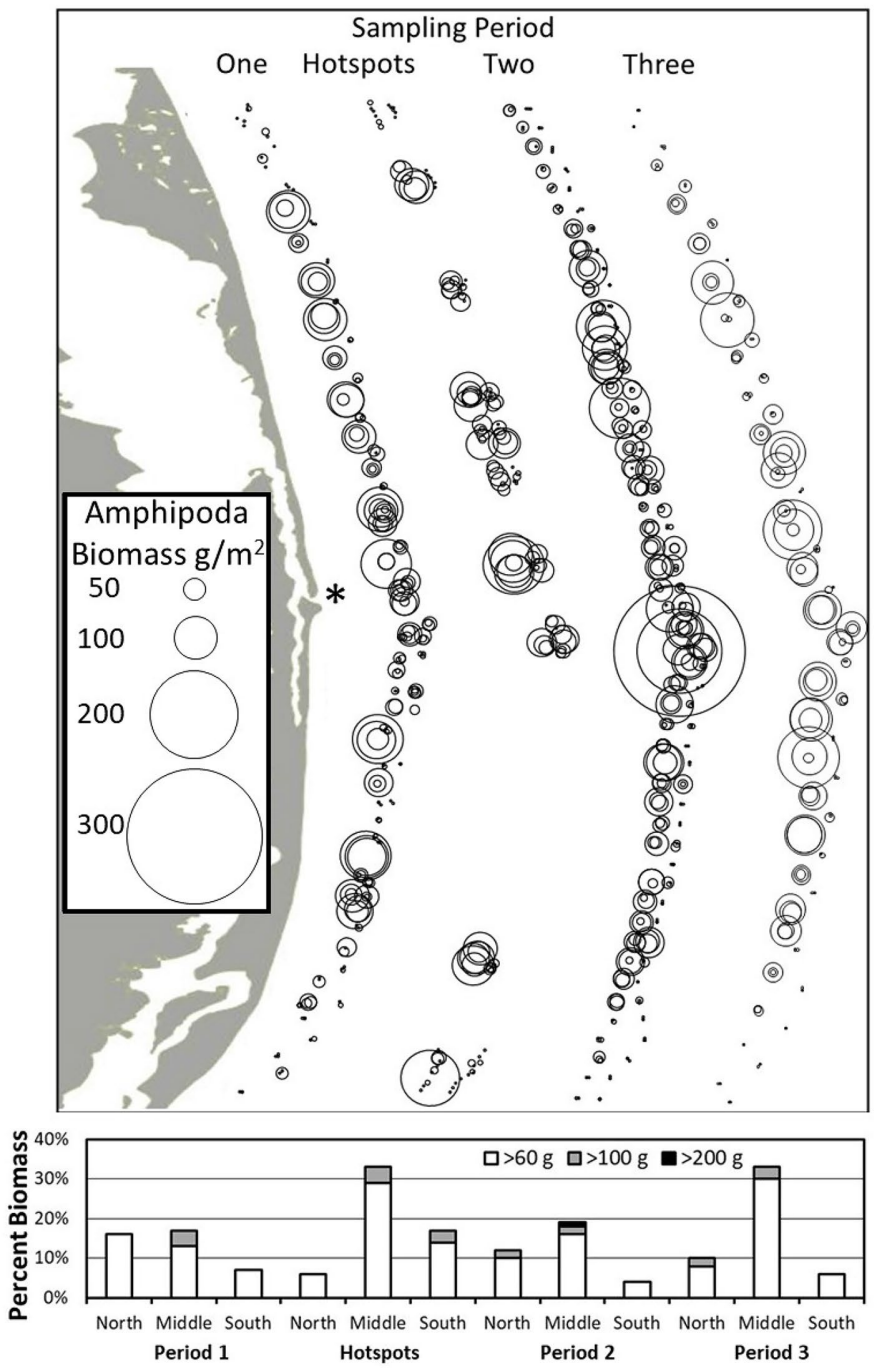
Amphipoda wet tissue biomass (g/m^2^) by sampling period in the 2015 nearshore study area. Bubbles represent replicate values. The maximum observed biomass of ~ 300 g/m^2^ was collected in Period 2 from the middle zone in 12.5 m water depth and medium sand. The Sakhalin Island shoreline is provided for context with * marking the mouth of Piltun Bay. The bar chart of amphipod biomass at the bottom presents the proportions of amphipod wet weight biomass above 60, 100, and 200 g/m^2^

Focusing more closely on Amphipoda, biomass concentrations demonstrated strong spatial–temporal variations. Amphipoda biomass was highest in the North and Middle Zones of the detailed sampling grid area during sampling Period 1 with 16% and 17% of biomass values greater than 60 g/m^2^ (Fig. 3). Biomass concentrations were lower in the South Zone than the other zones (only 7% of values were > 60 g/m^2^) in Period 1. In sampling Period 2, the distribution of Amphipoda biomass was highest in the Middle Zone with 19% of biomass values greater than 60 g/m^2^. Twelve percent of biomass values were greater than 60 g/m^2^ in the North Zone and 4% in the South Zone in Period 2. Biomass was greater in the middle portion of the study area in sampling Period 3 with 33%, 10%, and 6% of biomass values greater than 60 g/m^2^ in the Middle, North, and South Zones, respectively. Thirty-three percent of values were greater than 60 g/m^2^ in Middle Zone feeding hotspots. Overall, 11% of the sampling area had biomass values greater than 60 g/m^2^, 2% greater than 100 g/m^2^, and one sample was > 200 g/m^2^, representing the highest biomass concentrations (maximum = 296 g/m^2^) occurring in the Middle Zone and adjacent to the mouth of Piltun Bay in sampling Period 2.

**Fig. 4:**
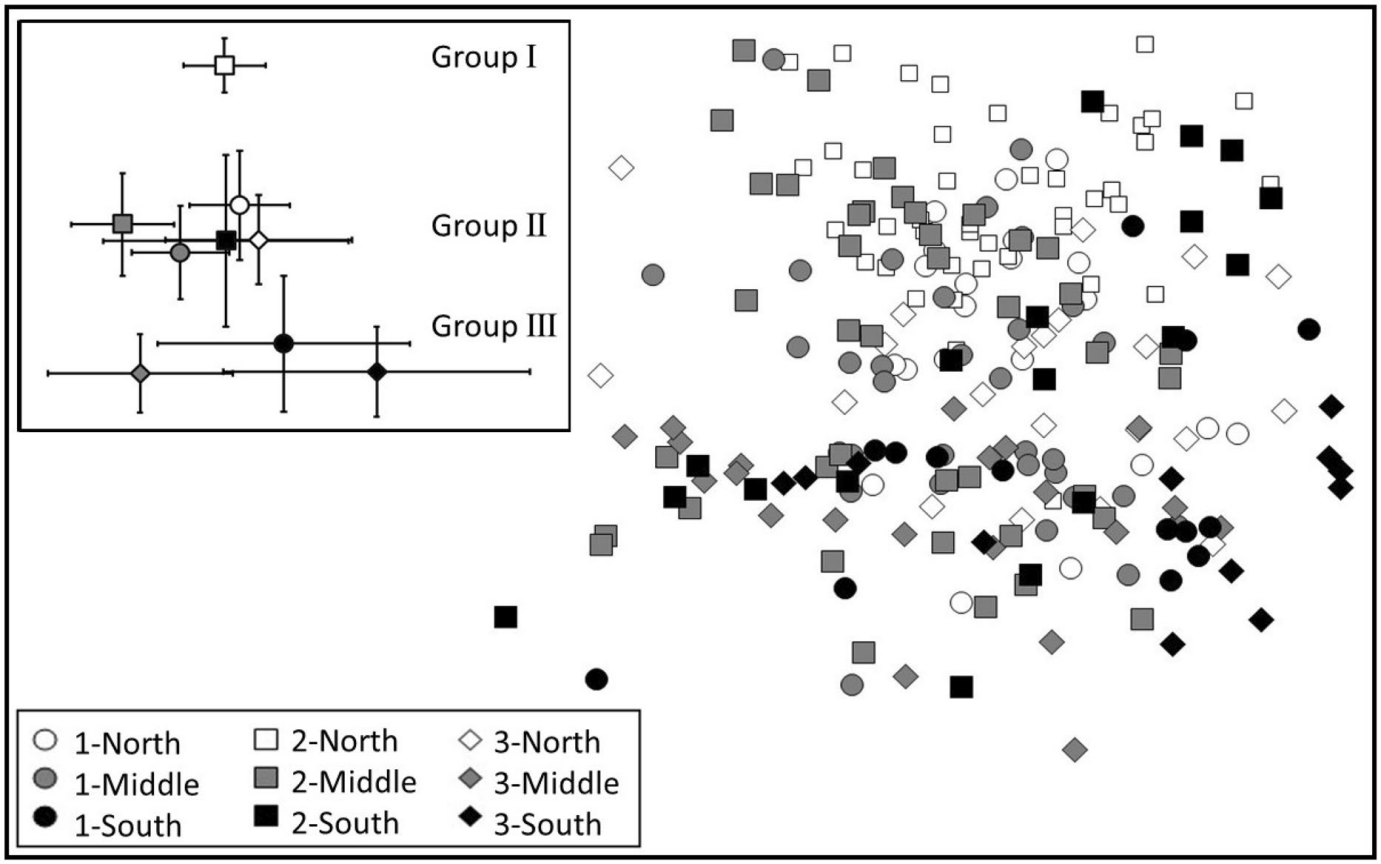
Nonmetric multidimensional scaling of wet tissue biomass for the 2015 detailed grid for Periods 1–3 at North, Middle, and South Zones. The stations are symbolized by period and zone. Centroids and 95% confidence intervals for the period by zone combinations are presented in the inset. MDS station groups are indicated in the centroid plot

The MDS plot demonstrated substantial overlap among the period/zone observations (Fig. 4). Nevertheless, the centroid for the first group of stations (group 1: Period 2, North Zone) was well separated from other centroids (inset, Fig. 4). Group 2 included Periods 1 and 3 for the North Zone, Periods 1 and 2 in the Middle Zone, and Period 2 in the South Zone, with this group’s centroids positioned in the middle of the plot. Group 3 consisted of Period 3 from the Middle Zone and the South Zone in Periods 1 and 3, with the centroids in the bottom of the plot. Centroid confidence intervals demonstrated low variability for Group 1 and notably higher variability for period-zone combinations in Group 3 along the horizontal axis.

**Fig. 5:**
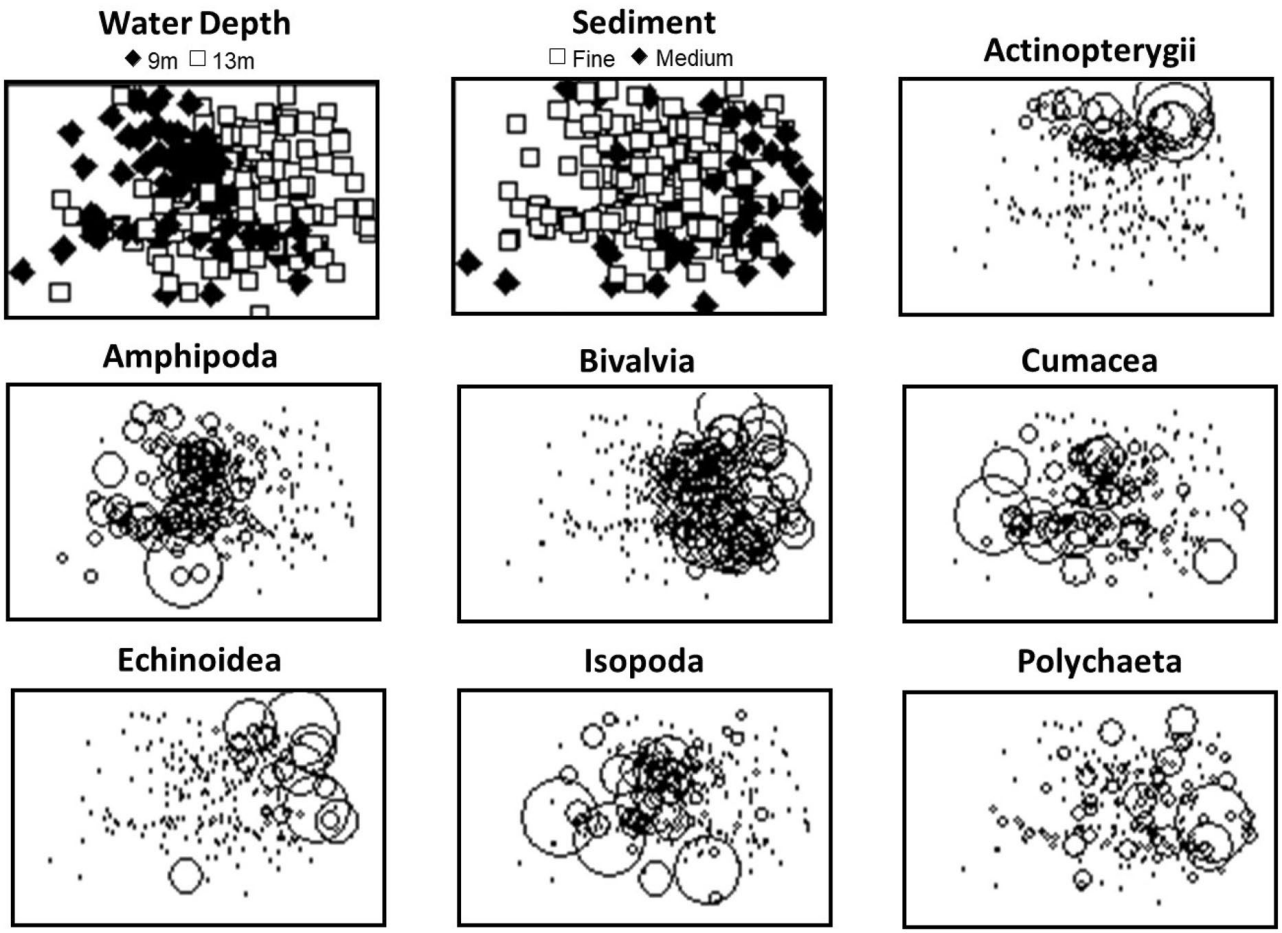
Overlays of depth and sediment categories and dominant invertebrate and vertebrate wet tissue biomass on the MDS ordination for the 2015 detailed grid. Bubbles represent the biomass with larger bubbles indicating higher biomass

The ordination reflects structuring by depth and sediment characteristics with stations having fine sand in 9 m water depth positioned to the right in the plot and medium sand in 13 m depth to the left (Fig. 5). Bubble plots of biomass indicated that the MDS ordination largely reflected the distributions of Amphipoda that were highest in fine sand and shallower water (large bubbles to the left in the MDS ordination overlay) and Bivalvia with higher biomass in medium sand and deeper water (larger bubbles to the right; Fig. 5). Rankings of taxon categories by biomass further demonstrated that amphipods and bivalves had the highest biomass except for Period 2 in the North Zone, where Actinopterygii had the second highest biomass (Table 2). Other taxa, including Actinopterygii and Isopoda, were periodically high, as reflected by large bubbles in the top left of the bubble plot for Actinopterygii in deeper water and towards the bottom right for Isopoda in shallower water (Fig. 5). Echinoidea demonstrated strong spatial patterning in the MDS plot with high values in deeper water and medium sand. Patterns for Cumacea and Polychaeta were less clear but higher biomass for Cumacea tended to be in shallow water and in deeper water for Polychaeta.

**Table 2:** Ranking of numerically dominant benthic groups (five groups with highest wet tissue biomass) by period and zone for the 2015 detailed grid

	North			Middle			South		
Period	Taxon	Biomass	SD	Taxon	Biomass	SD	Taxon	Biomass	SD
1	Bivalvia	59.08	68.13	Bivalvia	66.44	62.67	Bivalvia	92.19	65.04
	Amphipoda	19.97	18.02	Amphipoda	31.79	20.98	Amphipoda	19.24	16.56
	Actinopterygii	8.14	9.12	Isopoda	8.30	9.18	Polychaeta	4.33	4.43
	Polychaeta	7.64	11.84	Actinopterygii	5.82	12.16	Isopoda	1.85	2.34
	Isopoda	6.32	2.84	Polychaeta	3.97	2.95	Ascidia	1.59	5.73
2	Bivalvia	69.13	53.66	Bivalvia	38.18	30.70	Bivalvia	58.20	38.07
	Actinopterygii	42.92	41.42	Amphipoda	30.90	37.87	Amphipoda	11.83	16.73
	Amphipoda	15.19	12.20	Actinopterygii	11.73	14.85	Actinopterygii	9.28	11.67
	Echinoidea	7.11	12.25	Isopoda	5.12	5.72	Echinoidea	8.60	14.31
	Polychaeta	6.08	5.60	Polychaeta	2.51	2.22	Polychaeta	3.17	1.80
3	Bivalvia	57.73	56.24	Bivalvia	40.08	44.88	Bivalvia	59.63	44.54
	Amphipoda	18.36	18.36	Amphipoda	36.68	30.68	Amphipoda	13.96	24.72
	Echinoidea	7.31	11.69	Isopoda	14.46	17.02	Polychaeta	7.81	3.86
	Polychaeta	5.76	4.16	Polychaeta	5.82	6.66	Echinoidea	5.82	9.64
	Isopoda	2.55	1.59	Echinoidea	1.86	5.35	Isopoda	1.46	2.62

*Biomas*s average biomass (g/m^2^) and *SD* standard deviation

NPMANOVA demonstrated significant differences by water depth and sediment type and a significant period by zone interaction (Table 3). The significant differences in community structure by water depth and sediment factor levels were apparent in the MDS overlays (Fig. 5; Table 3). Multiple comparisons for the period by zone interaction were largely aligned with the MDS centroid plot as well. The North Zone in Period 2 (MDS group 1 positioned in the upper portion of the plot of Fig. 4) was significantly different from all the other period/zone combinations. Comparing groups 2 and 3, Period 1 North Zone and Period 2 Middle Zone of MDS group 2 were significantly different than all group 3 period and zone combinations; group 2 Period 1 Middle Zone was significantly different than Period 3 South Zone in group 3; and Period 3 North Zone of group 2 was significantly different than Period 3 Middle Zone from group 3. Within-group differences were only apparent for MDS group 2 with the North Zone in Period 3 being significantly different than Periods 1 and 2 in the Middle Zone.

Table 3 NPMANOVA multiple comparisons of the period * zone interaction for the 2015 detailed grid. The Bray–Curtis similarity matrix used for the MDS ordination was the response matrix. Multiple comparisons are organized by MDS group. Bold indicates significant multiple comparisons (*p* ≤ 0.05) of period-zone combinations (P-Z). All main (period, sediment type, water depth, and zone) and interaction (period * zone) effects of the full NPMANOVA model were significant (*p* < 0.002). Periods are 1–3 and zones are N, M, and S for North, Middle, and South Zones.

## Discussion

### Spatial–temporal dynamics

Evidence for a seasonal progression of biomass was not strong for benthic communities adjacent to northeastern Sakhalin Island in 2015. As with many research efforts in ice-affected systems, sampling in the present study occurred after ice-out and after ice-edge and spring phytoplankton blooms. As a result, a clear seasonal pattern in benthic biomass was not observed as we sampled just prior to and during expected peaks (Maresh et al. 2022). Some differences were apparent in biomass, but these differences mostly represented negligible to small-sized effects: Amphipoda biomass increased in the middle zone as the season progressed but declined across seasons in the North and South Zones; Cumacea biomass was high in Period 3 in the Middle and South Zones; Isopoda biomass was highest in Period 3 in the Middle Zone; and Polychaeta biomass was highest in Period 3. Biomass values were often high in Period 1, as it was for Amphipoda in the North and South Zones, Bivalvia across all zones, Isopoda in the North Zone, and total prey biomass for the Middle and South Zones.

Amphipod biomass in the nearshore feeding area for 2015 was low compared to the temporal record, reflecting a long-term trend of declining biomass (Blanchard et al., 2019). Macro- to global-scale processes presumably contribute to long-term trends, possibly through control of primary production and advection of deposited primary production by summer coastal circulation driven by the Amur River, upwelling, winter water currents, and ice conditions (Blanchard, 2015; Blanchard et al., 2010, 2019; Cloern et al., 2010; Drinkwater et al., 2010; Lehtonen & Andersin, 1998; Rutenko & Sosnin, 2014).

### Sources of variability

Seasonal hydrographic characteristics of the northeastern Sakhalin Island coast that influence benthic communities within the nearshore feeding area include Amur River discharges, summer coastal upwellings, and water freshening from lagoons (Demchenko et al., 2016; Fadeev, 2013). Amur River discharges strengthen summer water column stratification and increase coastal water temperatures (Rutenko & Sosnin, 2014). Coupled with the frequent wind-driven upwelling of colder, nutrient-rich water, summer stratification promoted by freshwater encourages production supporting nearshore benthic communities (Rutenko et al., 2009). The amphipod *M. affinis* responds directly to sedimentation of particulate organic carbon (POC) from marine primary production (Lehtonen & Andersin, 1998), a seasonal process controlled by climatic and oceanographic characteristics. Climate-controlled variations in discharge from the Amur River could be a significant influence on benthic community biomass at both short and long-term scales (Rutenko & Sosnin, 2014). Presumably, resuspension and lateral advection of POC in summer and under winter sea ice contribute to the high benthic biomass in the nearshore Sakhalin area, as also noted for the Chukchi Sea (Blanchard, 2015; Blanchard & Feder, 2014; Feder et al., 1994). Seasonal patterns of primary production and carbon availability are largely unknown for this region and the lack of information is a significant data gap for understanding benthic community dynamics.

The present study demonstrates that community structures differ among depth strata, despite the small 4-m difference. Amphipods have greater biomass in shallower waters (~ 9 m) and fine sand while bivalve biomass is greater in deeper water (~ 13 m) and medium sand. Biomass concentrations of other dominant fauna also vary with depth, with Actinopterygii and Echinoidea found in deeper waters, and with Isopoda in shallower waters. Cumacea and Polychaeta, however, were less predictable. Prior evidence from 2001 (Fadeev, 2002) indicates that amphipod biomass can be quite high in very shallow water depths (potentially ~ 2 times greater), where gray whale mother and calf pairs and juveniles are more commonly observed (~ ≤ 7 m water depth; Blanchard et al., 2019; Fadeev, 2002, 2007; Sychenko, 2011). Water depth was a significant factor in the distributions of other macrobenthic groups in ANCOVA as well, except for Actinopterygii and Isopoda. Depth had a stronger effect for bivalve biomass than other animals (a medium-sized effect as compared to negligible to small-sized effects) with higher biomass in deeper water; the 13–15 m depth range is suggested as optimal for bivalve biomass in the nearshore area (Sobolevskii et al., 2000). If related to circulation, current strengths, or wind-driven sediment instability, shifts in the conditions defining the optimal depth range may reduce or extend bivalve biomass distributions, and could be an indirect result from regional climatic drivers acting through water discharge from the Amur River and winds. Outflows of particulate organic carbon in summer from Piltun Bay likely contributed to spatial differences in biomass as well. Piltun Bay represents a point of change in seafloor and shoreline topography, so complexity and ecological change should be expected in that area.

The ME hypotheses demonstrated that temporal and spatial differences were small, with the exception of a medium-sized effect for Bivalvia. Criteria for ME hypotheses proposed by Blanchard et al. (2002) reflect natural variations as well as observed responses of benthic communities to anthropogenic disturbances. Benthic community characteristics can be highly variable and 20–30% changes in benthic community biomass and density are not uncommon. Thus, the small effect (*f* = 0.2, PV ~ 4%) reflects the naturally high variability of benthic communities, as compared to the small effect size (*f* = 0.1) of Cohen (1988) and Murphy et al. (2014). The medium-sized effect (*f* = 0.5, PV ~ 20%) was selected to represent changes that may indicate a community approaching or crossing an ecotone or ecological boundary (30–50% change). A 50% change or greater in biomass or density (*f* = 0.8, PV ~ 40%) is often associated with a disturbance event (Blanchard et al., 2002, 2003; De Grave & Whitaker, 1999) and was selected as the boundary for a large effect to capture major ecological change. The predominance of negligible to small effects in the present study demonstrates that changes in biomass across depths, zones, and sampling periods were within the range of natural variability and were not representative of major change for those groups, as compared to Blanchard et al. (2002). The medium-sized effect for Bivalvia indicates a larger gradient in biomass and a habitat-related pattern reflecting the ecological boundary for Bivalvia within the 9–13 m depth range. The value of the ME hypothesis grows with more varied applications and the usefulness of the approach lies in refining appropriate effect sizes across multiple environments.

### Prey biomass

Minimum amphipod biomass associated with gray whale feeding area is ~ 60–85 g/m^2^ wet weight biomass (Blanchard & Feder, 2014; Blanchard et al., 2013; Brower et al., 2017). Amphipod biomass was 60 g/m^2^ or higher in an area of 22 km^2^ of the 200 km^2^ nearshore Sakhalin Island study area, but total biomass, ranging from average values of ~ 70 to > 120 g/m^2^, appears to be high enough to represent gray whale feeding habitat in much of the study area where amphipod biomass was somewhat low but other crustaceans, polychaetes, and sand lance occurred with higher biomass. Energetic declines in ampeliscid amphipod populations of the northeastern Bering Sea might have resulted in increased eastern gray whale mortality in 1999–2000 (Coyle et al., 2007; Moore, 2008; Moore et al., 2003). It is not clear, however, that the 1999–2000 mortality event for the eastern population was in fact a response to a loss of prey or if it was due to other factors as the western gray whale population also experienced increased mortality in 1999–2000, while biomass in the Sakhalin Island feeding area was high in 2001 (Gailey et al., 2020; Salvadeo et al., 2015; Weller et al., 2002). Re-analysis of gray whale/benthic biomass relationships using comprehensive prey data (i.e., multiple prey classes) would be helpful to discern how total prey biomass influences gray whales throughout their range. Integrated long-term studies incorporating multi-prey data are needed to address questions at temporal scales appropriate for climatic, macrobenthic, and gray whale population inferences throughout the whale’s range (Blanchard et al., 2019; IUCN, 2019).

## Supplementary Information

Below is the link to the electronic supplementary material.

Supplementary file1 (DOCX 35 kb)
