# Supplementary material for: Benthic studies adjacent to Sakhalin Island, Russia, 2015 I: benthic biomass and community structure in the nearshore gray whale feeding area
Source: Environ Monit Assess. 2022 Oct 18;194(Suppl 1):743. doi: 10.1007/s10661-022-10017-8 (PMC9579072; doi:10.1007/s10661-022-10017-8)
Supplement: Supplementary file 1 — Supplementary file1 (DOCX 35 kb) [file 10661_2022_10017_MOESM1_ESM.docx]

**Supplementary Material Appendix A**

**Table A-1** Wet biomass of dominant macrobenthic faunal categories (average g/m^2^) based on station averages of the 2015 detailed grid sampling zones and periods, Sakhalin Island, Russia. Total = biomass of Actinopterygii, Amphipoda, Bivalvia, Cumacea, Isopoda, and Polychaeta. The number of stations used for each zone and period were Period 1, Zone N = 22, Zone M = 32, and Zone S = 14 stations; for Period 2, Zone N = 42, Zone M = 41, and Zone S = 18 stations; and for Period 3, Zone N = 21, Zone M = 21, and Zone S = 12 stations. Actinopt. = Actinopterygii

Period 1 Period 2 Period 3

| Group | Zone | Mean | SD | 95% CI | Mean | | SD | 95% CI | | Mean | SD | 95% CI |
| --- | --- | --- | --- | --- | --- | --- | --- | --- | --- | --- | --- | --- |
| Actinopt. | N | 7.6 | 8.0 | (4, 11.3) | 38.3 | | 34.2 | (27.7, 49) | | 1.9 | 1.8 | (1, 2.7) |
|  | M | 5.3 | 11.1 | (1.3, 9.3) | 11.4 | | 13.4 | (7.1, 15.6) | | 0.1 | 0.3 | (-0.1, 0.2) |
|  | S | 1.3 | 3.7 | (-0.9, 3.4) | 5.7 | | 10.1 | (0.7, 10.7) | | 0.1 | 0.3 | (-0.1, 0.4) |
| Amphipoda | N | 28.9 | 24.6 | (17.7, 40.1) | 27.2 | | 21.7 | (20.5, 34) | | 24.3 | 19.1 | (15.6, 33) |
|  | M | 33.1 | 23.0 | (24.8, 41.4) | 36.0 | | 31.4 | (26.1, 45.9) | | 40.2 | 30.8 | (26.2, 54.2) |
|  | S | 22.0 | 18.5 | (11.3, 32.6) | 18.9 | | 18.8 | (9.6, 28.3) | | 14.7 | 20.4 | (1.8, 27.7) |
| Bivalvia | N | 55.8 | 57.5 | (29.6, 82) | 46.5 | | 49.1 | (31.2, 61.8) | | 53.0 | 50.4 | (30, 75.9) |
|  | M | 55.6 | 60.4 | (33.8, 77.4) | 24.9 | | 29.5 | (15.6, 34.2) | | 35.7 | 43.0 | (16.2, 55.3) |
|  | S | 80.5 | 65.4 | (42.7, 118.3) | 39.3 | | 39.7 | (19.5, 59.1) | | 48.0 | 40.4 | (22.3, 73.6) |
| Cumacea | N | 0.9 | 0.9 | (0.5, 1.3) | 0.3 | | 0.3 | (0.2, 0.4) | | 0.2 | 0.5 | (-0.1, 0.4) |
|  | M | 1.1 | 1.0 | (0.7, 1.4) | 0.7 | | 0.6 | (0.5, 0.9) | | 1.0 | 1.6 | (0.3, 1.8) |
|  | S | 0.3 | 0.4 | (0.1, 0.6) | 0.6 | | 0.6 | (0.3, 0.9) | | 0.8 | 1.0 | (0.2, 1.5) |
| Echinoidea | N | 1.7 | 3.3 | (0.2, 3.2) | 4.1 | | 9.8 | (1, 7.1) | | 4.9 | 10.1 | (0.3, 9.5) |
|  | M | 0.2 | 0.7 | (0, 0.5) | 0.0 | | 0.0 | (0, 0) | | 1.6 | 5.0 | (-0.7, 3.9) |
|  | S | 1.2 | 4.2 | (-1.3, 3.6) | 5.3 | | 11.8 | (-0.6, 11.1) | | 3.9 | 8.2 | (-1.3, 9.1) |
| Isopoda | N | 9.6 | 6.9 | (6.4, 12.7) | 4.0 | | 2.4 | (3.3, 4.8) | | 2.1 | 1.5 | (1.5, 2.8) |
|  | M | 9.1 | 11.0 | (5.1, 13.1) | 5.6 | | 6.1 | (3.7, 7.5) | | 12.6 | 16.0 | (5.3, 19.9) |
|  | S | 2.3 | 3.5 | (0.2, 4.3) | 1.6 | | 1.2 | (1, 2.2) | | 1.5 | 2.2 | (0.1, 2.9) |
| Polychaeta | N | 6.3 | 10.1 | (1.7, 10.9) | 5.0 | | 4.9 | (3.5, 6.5) | | 7.1 | 5.8 | (4.5, 9.7) |
|  | M | 3.8 | 2.8 | (2.8, 4.8) | 2.3 | | 2.0 | (1.7, 2.9) | | 5.5 | 6.3 | (2.6, 8.3) |
|  | S | 3.2 | 2.2 | (2, 4.5) | 2.6 | | 1.8 | (1.7, 3.6) | | 6.8 | 2.8 | (5, 8.6) |
| Total | N | 109.1 | 60.0 | (81.8, 136.4) | 121.4 | 68.3 | | (100.1, 142.7) | 88.6 | | 50.3 | (65.7, 111.5) |
|  | M | 107.9 | 54.5 | (88.3, 127.6) | 80.9 | 40.7 | | (68, 93.7) | 95.1 | | 37.4 | (78.1, 112.1) |
|  | S | 109.6 | 54.0 | (78.4, 140.7) | 68.7 | 42.4 | | (47.6, 89.8) | 71.9 | | 29.7 | (53.1, 90.8) |

**Table A-2** Biomass (g/m^2^) for seven macrobenthic faunal categories and total biomass from feeding hotspots by sampling zone in the nearshore gray whale feeding area in 2015, Sakhalin Island, Russia. The number of feeding hotspots sampled in each zone were N = 6, M = 2, and S = 3. Total = biomass of Actinopterygii, Amphipoda, Bivalvia, Cumacea, Isopoda, and Polychaeta

| Group | Zone | Mean | SD | 95% CI |
| --- | --- | --- | --- | --- |
| Actinopterygii | N | 11.5 | 8.5 | (8, 14.9) |
|  | M | 11.0 | 14.7 | (0.6, 21.4) |
|  | S | 0.2 | 0.4 | (0, 0.5) |
| Amphipoda | N | 28.8 | 12.4 | (23.7, 33.8) |
|  | M | 51.8 | 15.4 | (40.9, 62.6) |
|  | S | 26.6 | 18.4 | (16, 37.2) |
| Bivalvia | N | 39.5 | 22.9 | (30.1, 48.8) |
|  | M | 43.5 | 41.4 | (14.3, 72.8) |
|  | S | 82.0 | 52.0 | (51.9, 112) |
| Cumacea | N | 0.5 | 0.2 | (0.4, 0.6) |
|  | M | 0.6 | 0.1 | (0.6, 0.7) |
|  | S | 0.4 | 0.3 | (0.3, 0.6) |
| Echinoidea | N | 7.9 | 12.6 | (2.7, 13) |
|  | M | 0.2 | 0.3 | (0, 0.5) |
|  | S | 2.0 | 3.1 | (0.2, 3.7) |
| Isopoda | N | 12.5 | 4.1 | (10.8, 14.2) |
|  | M | 14.5 | 5.0 | (11, 18) |
|  | S | 1.9 | 1.4 | (1, 2.7) |
| Polychaeta | N | 2.8 | 1.9 | (2, 3.6) |
|  | M | 7.3 | 4.6 | (4, 10.5) |
|  | S | 3.7 | 1.2 | (3, 4.4) |
| Total | N | 95.5 | 16.1 | (89, 102.1) |
|  | M | 128.7 | 40.3 | (100.2, 157.2) |
|  | S | 114.8 | 38.6 | (92.5, 137.1) |

**Table A-3** Degrees of freedom and noncentrality parameters for the power analysis based on the ANCOVA for the 2015 detailed grid. N DF = numerator degrees of freedom, Den DF = denominator (error) degrees of freedom, λ_C_ = noncentrality parameter for the effect-size of *f* = 0.1 as proposed by Cohen (1988) and λ_S_ = noncentrality parameter for the small effect-size of *f* = 0.2, λ_M_ = noncentrality parameter for the medium effect-size of *f* = 0.5, and λ_L_ = noncentrality parameter for the large effect-size of *f* = 0.8 proposed by Blanchard et al. (2002). The F-value of 2 is the median for the depth^2^ effect of the ANCOVA, F = 4 is near the median for the Z:P interaction (5.6), F = 8 is near the median for the period and zone effects (7.6 and 8.6), and F = 16 is the median for the depth effect (16.4). P_null_ = the p-values for the F-values under the standard null hypothesis. The noncentrality parameters were calculated as λ = Den DF*f/(1-f), as suggested by Murphy and Myors (2014)

|  |  |  |  |  |  |  | F = 2 | F = 4 | F = 8 | F = 16 |
| --- | --- | --- | --- | --- | --- | --- | --- | --- | --- | --- |
| Effect | N DF | Den DF | λ_C_ | λ_S_ | λ_M_ | λ_L_ | P_null_ | P_null_ | P_null_ | P_null_ |
| Zone (Z) | 2 | 102 | 1.0 | 4.1 | 25.5 | 39.8 | 0.141 | 0.021 | 0.001 | 0.000 |
| Period (P) | 2 | 639 | 6.5 | 25.6 | 159.8 | 249.3 | 0.136 | 0.019 | 0.000 | 0.000 |
| Z:P | 4 | 633 | 6.4 | 25.4 | 158.3 | 247.0 | 0.093 | 0.003 | 0.000 | 0.000 |
| Depth | 1 | 215 | 2.2 | 8.6 | 53.8 | 83.9 | 0.159 | 0.047 | 0.005 | 0.000 |
| Depth^2^ | 1 | 633 | 6.4 | 25.4 | 158.3 | 247.0 | 0.158 | 0.046 | 0.005 | 0.000 |

**Table A-4** Power of selected F-values against the noncentrality parameters for the effect-sizes proposed by Cohen (1988) and Blanchard et al. (2002) for ANCOVA of biomass for the 2015 detailed grid. The F-value of 2 is the median for the depth^2^ effect of the ANCOVA, F = 4 is near the median for the Z:P interaction (5.6), F = 8 is near the median for the period and zone effects (7.6 and 8.6), and F = 16 is the median for the depth effect (16.4). Pc = power of the F-value for the noncentral F associated with *f* = 0.1 of Cohen (1988) and P_S_ = power of the F-value for the noncentral F associated with *f* = 0.2, P_M_ = power of the F-value for the noncentral F associated with *f* = 0.5, and P_L_ = power of the F-value for the noncentral F associated with *f* = 0.8 of Blanchard et al. (2002). Power is generally low for all effects and effect-sizes for F = 2 except that power is moderate (p > 0.7) for the effect-size of *f* = 0.1 (a value proposed by Cohen, 1988) for zone. Power is moderate to high for *f* = 0.1 for zone, the zone*period interaction, and depth for F = 4. For F = 8, power is high (p > 0.8) for all ANCOVA effects for *f* = 0.1 except depth^2^ and high for the small effect of *f* = 0.2 for zone. Power is high for all ANCOVA effects for *f* = 0.1 and moderate to high for the small effect of *f* = 0.2 for zone, the zone*period interaction, and depth

|  | F = 2 |  |  |  | F = 4 |  |  |  |
| --- | --- | --- | --- | --- | --- | --- | --- | --- |
| Effect | Pc | Ps | Pm | Pl | Pc | Ps | Pm | Pl |
| Zone | **0.72** | 0.39 | 0.00 | 0.00 | **0.93** | **0.72** | 0.01 | 0.00 |
| Period | 0.22 | 0.00 | 0.00 | 0.00 | 0.54 | 0.01 | 0.00 | 0.00 |
| Z:P | 0.39 | 0.00 | 0.00 | 0.00 | **0.84** | 0.09 | 0.00 | 0.00 |
| Depth | 0.47 | 0.06 | 0.00 | 0.00 | **0.70** | 0.18 | 0.00 | 0.00 |
| Depth^2^ | 0.13 | 0.00 | 0.00 | 0.00 | 0.30 | 0.00 | 0.00 | 0.00 |
|  |  |  |  |  |  |  |  |  |
|  | F = 8 |  |  |  | F = 16 |  |  |  |
| Effect | Pc | Ps | Pm | Pl | Pss | Ps | Pm | Pl |
| Zone | **1.00** | **0.96** | 0.13 | 0.01 | **1.00** | **1.00** | 0.68 | 0.24 |
| Period | **0.90** | 0.12 | 0.00 | 0.00 | **1.00** | 0.69 | 0.00 | 0.00 |
| Z:P | **1.00** | 0.63 | 0.00 | 0.00 | **1.00** | **1.00** | 0.00 | 0.00 |
| Depth | **0.91** | 0.46 | 0.00 | 0.00 | **0.99** | **0.85** | 0.00 | 0.00 |
| Depth^2^ | 0.62 | 0.01 | 0.00 | 0.00 | **0.93** | 0.15 | 0.00 | 0.00 |

**Table A-5** ANCOVA of ln(X+1)-transformed biomass for seven faunal cateogries and total biomass for the 2015 detailed grid. P-values in bold are significant at α = 0.05. N DF = numerator and D DF = denominator (error) degrees of freedom, F = observed F-statistic and Pnull = p-value for null hypothesis. F-critical values and p-values are presented for ME hypotheses for small (Fs and Ps), medium (F_M_ and P_M_), and large-sized (F_L_ and P_L_) effects. Actinopt. = Actinopterygii and Amph. = Amphipoda. Denominator degrees of freedom were determined using the Satterthwaite approximation from mixed models

| Group | Effect | N DF | D DF | F | P_Null_ | Fs | Ps | F_M_ | P_M_ | F_L_ | P_L_ |
| --- | --- | --- | --- | --- | --- | --- | --- | --- | --- | --- | --- |
| Actinopt. | Zone (Z) | 2 | 79 | 14.9 | **<0.0001** | 6.86 | **0.0006** | 20.50 | 0.2043 | 42.63 | 0.9444 |
|  | Period (P) | 2 | 643 | 52.1 | **<0.0001** | 23.36 | **0.0000** | 104.99 | 0.9922 | 247.38 | 1.0000 |
|  | Z:P | 4 | 636 | 8.2 | **<0.0001** | 12.17 | 0.3444 | 52.55 | 1.0000 | 123.02 | 1.0000 |
|  | Depth (D) | 1 | 139 | 1.0 | 0.3239 | 16.50 | 0.9153 | 59.74 | 1.0000 | 131.26 | 1.0000 |
|  | D^2^ | 1 | 549 | 2.1 | 0.1497 | 40.51 | 0.9994 | 182.08 | 1.0000 | 427.83 | 1.0000 |
| Amph. | Z | 2 | 100 | 11.6 | **<0.0001** | 7.61 | **0.0063** | 24.06 | 0.6137 | 51.01 | 0.9990 |
|  | P | 2 | 615 | 4.5 | **0.0116** | 22.62 | 0.9817 | 101.07 | 1.0000 | 237.73 | 1.0000 |
|  | Z:P | 4 | 601 | 3.5 | **0.0079** | 11.72 | 0.9356 | 50.11 | 1.0000 | 117.01 | 1.0000 |
|  | D | 1 | 324 | 73.3 | **<0.0001** | 27.93 | **0.0000** | 116.45 | 0.6618 | 267.66 | 1.0000 |
|  | D^2^ | 1 | 655 | 27.1 | **<0.0001** | 46.19 | 0.4673 | 212.40 | 1.0000 | 502.33 | 1.0000 |
| Bivalvia | Z | 2 | 107 | 3.5 | **0.0353** | 7.85 | 0.3737 | 25.21 | 0.9959 | 53.73 | 1.0000 |
|  | P | 2 | 638 | 14.4 | **<0.0001** | 23.24 | 0.4132 | 104.36 | 1.0000 | 245.83 | 1.0000 |
|  | Z:P | 4 | 629 | 3.9 | **0.0036** | 12.09 | 0.9164 | 52.09 | 1.0000 | 121.88 | 1.0000 |
|  | D | 1 | 224 | 134.8 | **<0.0001** | 21.93 | **0.0000** | 86.20 | **0.0001** | 194.52 | 0.6266 |
|  | D^2^ | 1 | 640 | 0.1 | 0.7282 | 45.39 | 1.0000 | 208.04 | 1.0000 | 491.60 | 1.0000 |
| Cumacea | Z | 2 | 104 | 11.1 | **<0.0001** | 7.75 | **0.0090** | 24.74 | 0.6867 | 52.63 | 0.9996 |
|  | P | 2 | 641 | 0.5 | 0.5969 | 23.31 | 1.0000 | 104.70 | 1.0000 | 246.68 | 1.0000 |
|  | Z:P | 4 | 632 | 12.4 | **<0.0001** | 12.13 | **0.0428** | 52.32 | 1.0000 | 122.45 | 1.0000 |
|  | D | 1 | 206 | 2.2 | 0.1354 | 20.79 | 0.9143 | 80.59 | 1.0000 | 181.06 | 1.0000 |
|  | D^2^ | 1 | 626 | 42.3 | **<0.0001** | 44.66 | 0.0711 | 204.14 | 1.0000 | 482.05 | 1.0000 |
| Echinoidea | Z | 2 | 109 | 6.3 | **0.0027** | 7.90 | 0.1115 | 25.49 | 0.9602 | 54.40 | 1.0000 |
|  | P | 4 | 622 | 1.6 | 0.1835 | 23.09 | 0.9876 | 103.59 | 1.0000 | 243.94 | 1.0000 |
|  | Z:P | 2 | 633 | 4.2 | **0.0148** | 11.99 | 0.9982 | 51.60 | 1.0000 | 120.67 | 1.0000 |
|  | D | 1 | 255 | 41.3 | **<0.0001** | 23.86 | **0.0010** | 95.84 | 0.9347 | 217.76 | 1.0000 |
|  | D^2^ | 1 | 653 | 7.5 | **0.0063** | 46.12 | 0.9911 | 211.98 | 1.0000 | 501.31 | 1.0000 |
| Isopoda | Z | 2 | 107 | 22.1 | **<0.0001** | 7.84 | **0.0000** | 25.20 | 0.1053 | 53.71 | 0.9397 |
|  | P | 2 | 632 | 8.2 | **0.0003** | 23.09 | 0.8641 | 103.55 | 1.0000 | 243.85 | 1.0000 |
|  | Z:P | 4 | 622 | 19.8 | **<0.0001** | 11.99 | **0.0002** | 51.57 | 0.9998 | 120.61 | 1.0000 |
|  | D | 1 | 251 | 0.7 | 0.4182 | 23.60 | 0.9909 | 94.55 | 1.0000 | 214.64 | 1.0000 |
|  | D^2^ | 1 | 653 | 1.8 | 0.1748 | 46.10 | 0.9999 | 211.86 | 1.0000 | 501.01 | 1.0000 |
| Polychaeta | Z | 2 | 86 | 4.3 | **0.0171** | 7.13 | 0.2090 | 21.74 | 0.9658 | 45.58 | 1.0000 |
|  | P | 2 | 647 | 15.4 | **<0.0001** | 23.48 | 0.3608 | 105.63 | 1.0000 | 248.96 | 1.0000 |
|  | Z:P | 4 | 643 | 2.4 | **0.0502** | 12.26 | 0.9908 | 53.05 | 1.0000 | 124.25 | 1.0000 |
|  | D | 1 | 141 | 16.8 | **0.0001** | 16.58 | **0.0469** | 60.10 | 0.9623 | 132.11 | 1.0000 |
|  | D^2^ | 1 | 514 | 0.8 | 0.3767 | 38.63 | 0.9999 | 172.11 | 1.0000 | 403.41 | 1.0000 |
| Total | Z | 2 | 97 | 3.8 | **0.0266** | 7.48 | 0.2947 | 23.46 | 0.9882 | 49.61 | 1.0000 |
|  | P | 2 | 644 | 7.0 | **0.0010** | 23.39 | 0.9246 | 105.16 | 1.0000 | 247.80 | 1.0000 |
|  | Z:P | 4 | 637 | 7.0 | **<0.0001** | 12.19 | 0.5204 | 52.65 | 1.0000 | 123.28 | 1.0000 |
|  | D | 1 | 174 | 15.9 | **0.0001** | 18.81 | 0.0949 | 70.86 | 0.9948 | 157.77 | 1.0000 |
|  | D^2^ | 1 | 589 | 0.1 | 0.8119 | 42.69 | 1.0000 | 193.66 | 1.0000 | 456.27 | 1.0000 |
